# Understanding the impact of an online post-ICU peer support group: A survey of patients and families

**DOI:** 10.1177/17511437251381958

**Published:** 2025-11-02

**Authors:** Natalie McEvoy, Sabina Mason, Serena O’Brien, Barbara Egan, Melanie Ryberg

**Affiliations:** 1 Royal College of Surgeons in Ireland, Dublin, Ireland; 2 Tallaght University Hospital, Dublin, Ireland; 3 ICUsteps Dublin, Dublin, Ireland; 4 Mater Misericordiae University Hospital, Dublin, Ireland; 5 University College Dublin, Dublin, Ireland; 6 Trinity College Dublin, Dublin Ireland

**Keywords:** peer support, critical illness, critical care, ICU survivorship, post-intensive care syndrome

## Abstract

Peer support groups for Intensive Care Unit (ICU) survivors and family members hold promising potential to reduce the psychological burden associated with critical illness and increase social support. We invited all ICU survivors and family members who attended an online peer support group in Ireland (‘ICUsteps Dublin’ open to those across the Island of Ireland) over a five-year period to complete a brief satisfaction survey. Overall, respondents reported high levels of satisfaction. Four key themes emerged from the open-ended responses: decreased feeling of isolation, gaining insight and perspectives, shared experiences and suggestions for improvement.

## Introduction

Many ICU survivors and family members experience long-term physical, psychological and social challenges which may persist for months or years following discharge.^1,2^ However, they frequently report attendance at peer support group meetings can help alleviate some of these issues.^3^ This study aimed to assess satisfaction with an online peer support group, and to identify opportunities to enhance physical and psychological recovery through improved support during these sessions via an anonymous survey. Meetings are peer-led, last 90 min and are facilitated by volunteers including ICU nurses.

## Methods

Seventy-two participants who had attended this online post-ICU peer support group on any occasion between March 2020 and March 2025 were contacted by email with an invitation to participate; this included a participant information leaflet to ensure informed consent, which was recorded electronically via the digital survey platform (SurveyMonkey). The survey included demographic questions, open and closed questions to characterise respondents’ experiences, as well as a validated measure of service satisfaction (Client Satisfaction Questionnaire; CSQ-8^4^). The study was approved by the Royal College of Surgeons in Ireland Research Ethics Committee (REC202504006). Quantitative responses were analysed descriptively; thematic analysis was used to analyse qualitative responses.^5^

## Results

The response rate was 58% (*N* = 42). Respondent demographics are presented in Table 1.

**Table 1.** Profile of survey respondents (*N* = 42).

ICU patients	81% (*n* = 34)	Family members	33% (*n* = 13)^a^
Age			
18–29 years, 5% (*n* = 2)	30–49 years, 29% (*n* = 12)	50–69 years, 45% (*n* = 19)	70+ years, 21% (*n* = 9)
Gender			
Female, 57% (*n* = 24)		Male, 43% (*n* = 18)	
Time between hospital discharge and first meeting^b^			
<3 months, 29% (*n* = 12)	3–6 months, 24% (*n* = 10)	6–12 months, 20% (*n* = 8)	>12 months, 27% (*n* = 11)
Number of meetings attended			
<5, 33% (*n* = 14)	5–10, 31% (*n* = 13)	10–20, 24% (*n* = 10)	>20, 12% (*n* = 5)

a Potential for overlap between groups acknowledged.

b Missing data not reported.

Most respondents rated the quality of the peer support group as excellent and would recommend it to a friend (*n* = 33; 85%). Seventy-one percent (*n* = 27) of respondents indicated that the meetings helped them to deal more effectively with their problems. Seventy-seven percent (*n* = 30) stated they would attend a meeting again in the future if needed.

Four key themes emerged:

### Theme 1: Decreased feeling of isolation

Many respondents reported that attending meetings led to feeling less isolated and expressed a profound sense of comfort in discovering they were not alone in their experiences:

*“Seeing that I was not alone in being a spouse of an ICU patient.”* Respondent 1

*“Knowing that I am not alone and that others have had similar experiences”* Respondent 19

### Theme 2: Gaining insights and perspectives

Respondents described how the meetings facilitated personal reflection and helped them better understand their own experiences, emotions, and recovery processes. Some realised aspects of their journey they hadn’t considered before, such as the emotional impact on loved ones:

*“It made me realise what my family may have gone through while I was ill, which I hadn’t thought of before now.”* Respondent 9

*“It made sense to a lot of things I had been going through, and the answers I wanted help with.”* Respondent 17

### Theme 3: Shared experiences

Participants described a sense of relief and reassurance in recognising that others had been through similar ICU experiences, which helped to normalise their emotional and psychological responses.

*“Being with people who could understand what I was experiencing, therefore giving me huge confidence that I was actually ‘normal’ and would get through this period of transition back to life.”* Respondent 5

Sharing stories with peers who “understood” was a powerful source of comfort and support.

*“That I wasn’t losing my mind. Hearing others talk about their experiences was like hearing myself speak.”* Respondent 30

### Theme 4: Suggestions for improvement

A number of suggestions for improvement were made including enhanced advertisement, increased frequency/face-to-face meetings, and additional input from healthcare professionals to present on topics so they could learn from others experiences.

*‘It was always interesting when the nurses or other knowledgeable members gave us their experience - maybe one 10 to 15 mins per session on a specific topic.”* Respondent 4

## Discussion

This survey highlights the value of online ICU peer support groups in the recovery phase post-critical illness for ICU survivors and their families. Overall, respondents reported high levels of satisfaction with the support received. Many identified benefits from shared experiences with peers, including comfort at being among others who “understood” without needing detailed explanation. Findings are consistent with previous studies suggesting that peer groups help survivors and family members make sense of their experiences through storytelling, empathy, and mutual support.^1,6^ The theme of feeling less isolated echoes previous findings, which suggest that ICU survivors often feel disconnected and misunderstood post-discharge, and that peer support can provide a sense of belonging.^7,8^ The theme of gaining insights and perspectives supports previous qualitative work showing that peer interactions help survivors make sense of their recovery trajectories and reduce psychological distress.^6^ Valuable suggestions for improvements were made.

This model could be explored and developed further by the health service to offer a tailored peer support group guided by experienced ICU professionals and former ICU patients and families, keeping support relevant at various stages of recovery.
